# [^18^F]FDG-PET reveals early postoperative cortical dysfunction after subthalamic nucleus deep brain stimulation in Parkinson’s disease

**DOI:** 10.1007/s00259-025-07552-0

**Published:** 2025-10-02

**Authors:** Christian Volz, Volker A. Coenen, Lars Frings, Michel Rijntjes, Horst Urbach, Philipp T. Meyer, Bastian E. A. Sajonz, Joachim Brumberg

**Affiliations:** 1 Department of Nuclear Medicine, Medical Center – University of Freiburg, Faculty of Medicine, University of Freiburg, Freiburg, Germany; 2 Department of Stereotactic and Functional Neurosurgery, Medical Center – University of Freiburg, Faculty of Medicine, University of Freiburg, Freiburg, Germany; 3 Department of Neurology and Clinical Neuroscience, Medical Center – University of Freiburg, Faculty of Medicine, University of Freiburg, Freiburg, Germany; 4 Department of Neuroradiology, Medical Center – University of Freiburg, Faculty of Medicine, University of Freiburg, Freiburg, Germany

**Keywords:** FDG PET, Deep brain stimulation, Parkinson’s disease, Subthalamic nucleus

## Abstract

**Purpose:**

Motor improvement and neuropsychiatric symptoms may immediately follow implantation of subthalamic nucleus deep brain stimulation (STN-DBS) electrodes in Parkinson’s disease (PD). We investigated the impact of electrode implantation prior to stimulation on transient dysfunction using cerebral [^18^F]fluorodeoxyglucose positron emission tomography ([^18^F]FDG-PET). Additionally, we sought to identify clinical and imaging variables associated with neuropsychiatric side effects (SE).

**Methods:**

This retrospective analysis included thirteen patients with advanced PD, receiving [^18^F]FDG-PET scans preoperatively and within three weeks (median 6 days) after STN-DBS surgery. We analyzed changes of regional metabolism in a voxel-wise manner and with volumes-of-interests. Moreover, we evaluated the extent and magnitude of metabolic decrease alongside the electrode trajectories. Neuropsychiatric SE were defined as novel, surgery-related neuropsychiatric symptoms listed in clinical records.

**Results:**

PET analyses revealed hypometabolism along the electrodes (mean magnitude up to -35.9 ± 18.0% and mean extent of 19.9 ± 10.2 ml per hemisphere) largely located in the frontal lobe (left: *k*_E_ = 1718 voxels; right: *k*_E_ = 1550 voxels, *p* < 0.05, false discovery rate corrected). Patients with neuropsychiatric SE were older (61 vs. 55 years; *p* = 0.043) and had lower normalized [^18^F]FDG uptake in the middle frontal gyrus (MFG) preoperatively (1.01 vs. 1.06; *p* = 0.030). Preoperative metabolism in the MFG and age were indicative of the occurrence of neuropsychiatric SE (area under the Receiver Operating Characteristic curve = 0.90).

**Conclusions:**

Reversible neuronal dysfunction after STN-DBS surgery can be detected by [^18^F]FDG-PET along the electrode trajectory. Preoperative MFG metabolism and age were associated with postoperative neuropsychiatric SE supporting the use of PET to improve patient selection and postoperative care concepts.

**Supplementary Information:**

The online version contains supplementary material available at 10.1007/s00259-025-07552-0.

## Introduction

Deep brain stimulation (DBS) of the subthalamic nucleus (STN) and globus pallidus internus (GPi) is a well-established, device-based therapy for the treatment of advanced Parkinson’s disease (PD), alleviating motor symptoms that are no longer adequately controlled by medication or reducing medication-related motor complications [1–3].

The actual stimulation via the electrodes is typically initiated within 2–12 weeks after stereotactic DBS electrode and stimulator implantation, although the timing varies between institutions [4]. In the early interval after surgery, however, effects on clinical symptoms are often observed without stimulation. The so-called “microlesion effect” (MLE) summarizes the rapid improvement of motor symptoms in the first days to weeks after surgery, before stimulation is initiated [4, 5]. The mechanisms and underlying principles of MLE remain incompletely understood [6]. However, it is believed to be indicative of accurate electrode placement and to reflect a local disruption of neuronal function in the target region, inducing sequential alterations in the basal ganglia network [7]. For instance, the STN provides excitatory glutamatergic input to the GPi and striatum [8].

Besides the pure motor effects, neuropsychiatric (including cognitive) side effects (SE) (e.g., delirium, verbal fluency (VF) or cognitive decline and mood disorders like hypomania) may occur during this period [9–11]. These neuropsychiatric SE cannot be reliably attributed to specific causes or etiological mechanisms [12, 13], but may impair quality of life [14]. Therefore, surgery-related factors or MLE are increasingly being considered as possible causes of neuropsychiatric SE [9, 11, 15]. Some studies have shown that decline of VF result from MLE in the STN or its projections [11, 15], whereas others identified parenchymal brain damage along the electrode trajectory due to surgery as an additional cause [16, 17]. Recent studies showed that white matter tract lesions on magnetic resonance imaging (MRI) (including projections to the frontal cortex) along the electrode trajectory after DBS surgery were associated with VF decline [18, 19]. The frontal cortex is involved in executive functions such as decision making, impulse control, and motor planning, as well as cognitive functions that are implicated in various neuropsychiatric disorders such as (hypo)mania, psychomotor slowing, impaired VF, cognitive impairment and delirium [20–23]. Therefore, it should be considered that neuropsychiatric SE following DBS surgery may be related to the impairment of frontal cortical-subcortical circuitries.

As the decision to initiate STN-DBS depends on its expected efficacy, short- and long-term outcome (including overall survival), it is important to elucidate and possibly prevent SE, e.g. by identifying predisposing factors. Besides demographic and clinical parameters, functional neuroimaging may be suitable for this purpose. Cerebral glucose metabolism measured with [^18^F]FDG-PET is an established method for assessing neuronal (dys)function and degeneration [24, 25]. High-resolution, fully-digital PET/CT systems, enable qualitative and quantitative assessment of small (sub)cortical brain regions and thus, in combination with [^18^F]FDG, more precise localization of metabolic changes [26, 27].

The aim of this study was to investigate alterations of regional cerebral glucose metabolism early after STN-DBS surgery compared to a baseline [^18^F]FDG-PET before surgery in patients with advanced PD. We hypothesized that DBS placement causes glucose hypometabolism witnessing neuronal dysfunction along the electrode trajectories. Furthermore, our second hypothesis proposed that extent and magnitude of hypometabolism (not only postoperatively, also possibly preoperatively) are associated with neuropsychiatric sequelae following STN-DBS surgery.

## Materials and methods

### Patients

This retrospective study included non-consecutive patients with advanced PD who were implanted with bilateral STN-DBS electrodes between May 2022 and November 2023 and underwent [^18^F]FDG-PET scans before surgery (baseline) and within three weeks postoperatively (follow-up) for clinical indications (differential diagnosis and therapy monitoring). Contraindications, including cognitive and neuropsychiatric deficits, were excluded through a multidisciplinary evaluation prior to STN-DBS. Patient records, based on routine clinical assessments by the treating neurologists and neurosurgeons, were reviewed for improvement of motor symptoms, reduction in Levodopa Equivalent Daily Dose (LED) and (sub-)acute neuropsychiatric (including cognitive) symptoms following DBS surgery, excluding alternative causes unrelated to the intervention. Based on the presence or absence of neuropsychiatric symptoms, patients were divided into two groups: one with SE, and one without SE. All patients provided written informed consent for STN-DBS, [^18^F]FDG-PET imaging, and retrospective data analysis. The local institutional review board approved the study (EK 21-1274).

### Implantation of DBS electrodes

A detailed description of our DBS electrode implantation procedure is provided elsewhere [28]. In brief, preliminary trajectories targeting the STN were planned on isotropic high resolution T2- and contrast enhanced T1-weighted MRI and adjusted based on intraoperative stereotactic computed tomography (CT) angiography to avoid vessels and sulci. Such trajectories typically traverse the MFG with an entry anterior to the coronal suture. A 14 mm burr hole was placed at the entry point of the trajectory and waxed to avoid osseous or epidural bleeding. A burr hole cover was fixed at the burr hole. Bipolar coagulation was performed prior to incision at the dural entry point followed by bipolar corticotomy. Two combined micro-macro-electrodes (Sonos Shielded Microelectrode, model STR-009080-00, Alpha-Omega, Israel) were lowered through guiding cannulas into the target region on each side with a Leksell G-frame (Elekta, Sweden). After electrophysiological and clinical target verification, DBS electrodes were implanted on the desired trajectory and anchored in the burr hole cover after fluoroscopic verification of implantation depth. Instead of combined micro-macro-electrodes a blunt-tip monopolar lesion electrode (Cosman, USA, or Inomed, Germany) was used on a single trajectory per side in one patient for clinical target verification. A postoperative CT was conducted directly after DBS electrode placement to verify the final electrode position and screen for intracerebral hemorrhage.

### PET acquisition

[^18^F]FDG-PET scans were performed using a fully-digital Vereos PET/CT system (Philips Healthcare, The Netherlands). After at least 6 h of fasting, 200 ± 29 MBq of [^18^F]FDG were injected intravenously under normoglycemic conditions, followed by standardized resting conditions. Fifty minutes after injection, a static PET scan was acquired over 10 min. We used low-dose CT (120 kV, 15 mAs) for attenuation correction and definition of electrode trajectory VOI. CT images were reconstructed using an iterative (standard brain kernel) with a voxel size of 1.17 × 1.17 × 2 mm³. Reconstruction of the corrected emission dataset was performed as previously described [26, 27].

### Image analysis

We performed a set of voxel-wise analyses (a-c) and volumes-of-interest (VOI) analyses (d) in Montreal Neurologic Institute (MNI) space by using statistical parametric mapping 12 (SPM12; www.fil.ion.ucl.ac.uk/spm) and MATLAB R2021b (Mathworks, USA). Furthermore, we evaluated the metabolism around the electrodes in the individual space (analysis e) with PMOD 4.3 (PMOD Technologies LLC, www.pmod.com/web). Preprocessing steps for analyses a-c were as follows: PET images were stereotactically normalized to an in-house [^18^F]FDG-PET template in MNI space, smoothed with a Gaussian filter (8 mm full width at half maximum; voxel size: 2 × 2 × 2 mm^3^), globally intensity-normalized and restricted to brain parenchyma by masking. (a) We performed a voxel-wise paired t-test to identify areas with reduced metabolism in the follow-up PET scans compared to baseline scans. (b) Baseline PET scans of patients with and without SE were each compared to thirteen scanner-matched healthy controls (54% male, 68.4 ± 6.8 years) from a prior study [29] using independent two-sample t-tests with age as covariate. For both analyses (a, b), statistical significance was defined at *p* < 0.001 (*k*_*E*_ ≥ 100 voxels), with at least peak voxels surviving false discovery rate (FDR) correction at *p* < 0.05 at voxel level. (c) To define the extent of metabolic changes, individual differences in normalized [^18^F]FDG uptake between baseline and follow-up PET were calculated with a paired voxel-by-voxel subtraction. Voxel-wise difference maps (follow-up PET – baseline PET) were transformed into Z-score maps using the global mean and standard deviation of the difference. The extent of metabolic decrease was defined as the number of voxels with Z-score ≤ −2. Non-frontal small clusters or single voxels were manually excluded to minimize unspecific effects. (d) The preprocessing steps of stereotactic normalization and smoothing in analysis d were identical to analyses a-c. Changes in regional [^18^F]FDG uptake baseline and follow-up PET were analyzed using predefined VOI and normalized to the mean uptake within a whole-brain parenchyma VOI. We selected the middle frontal gyrus (MFG) and putamen from the automated anatomical labeling atlas, version 3 [30] and the STN and GPi from the Human Motor Thalamus atlas [31] to represent possibly affected cortical and subcortical regions alongside the trajectory and the target region of DBS with their projections. (e) Last, the magnitude of metabolic decrease was assessed with manually generated individual VOI around the electrodes. Since follow-up PET/CT enables electrode localization, VOI delineation was based on CT and PET data (voxel size: 1 × 1 × 1 mm^3^). Baseline PET were registered to follow-up PET/CT. A grey matter VOI was generated from baseline PET (isocontur threshold 25% of the maximum intensity value). Electrode VOI were defined on CT (isodensity threshold 70% of the electrode´s Hounsfield-Units) extended 4 cm from the cortex entry and dilated 3-dimensionally (3, 6 and 9 voxels) to yield small, medium, and large sized trajectory VOI. The proportions of white matter and cerebrospinal fluid space were excluded using the grey matter VOI. Additionally, an adjusted grey matter VOI was created by subtracting large trajectory VOI. An example of trajectory and adjusted grey matter VOI contours is depicted in Supplementary Information 1. Normalized [^18^F]FDG uptake in the trajectory VOI was calculated relative to the adjusted grey matter VOI. Finally, we calculated the percentage change between baseline and follow-up PET.

### Statistical analyses

Statistical analyses were done with the software R (version 4.3.0; www.R-project.org) and GraphPad Prism9 (GraphPad Software; www.graphpad.com). Paired t-tests with FDR correction were used to compare the differences of normalized [^18^F]FDG uptake in the predefined VOI between baseline and follow-up PET (d). Pearson’s correlations (*r*, FDR-corrected) examined associations between percentage reductions in the predefined VOI. Two separate one-way repeated measures analysis of variance (RM-ANOVA) were conducted (one for each hemisphere) to assess differences in magnitude of metabolic decrease across three levels of trajectory VOI size (e). Group comparisons (patients with SE vs. patients without SE) of demographic and clinical characteristics (age, sex, symptom duration, Hoehn & Yahr stage, LED reduction) and image-based variables (extent and magnitude of metabolic decrease, MFG metabolism at baseline PET) were done using U-test statistics or χ² tests, as appropriate. Effect sizes were calculated using Cohen’s *d* for paired t-tests and rank-biserial correlation coefficient *r*_rb_ for U-test statistics. Receiver operating characteristics (ROC) curve analysis was performed to assess the ability of significant parameters to discriminate between the groups by calculating the area under the ROC curve (AUC). We fitted a multivariate logistic regression model to evaluate the combination of parameters.

## Results

### Patient characteristics

Thirteen patients met the inclusion criteria and were enrolled in this study (four women; median age: 59 years, range: 44–71 years). Median interval between baseline PET and DBS surgery was 125 (range: 1–786) days, while the median interval between surgery and follow-up PET was 6 (range: 5–16) days. Median PD symptom duration from onset to surgery was 7.5 (range: 2–15) years and the patients were in intermediate stages according to Hoehn & Yahr (stage 2: 38.5%, stage 3: 61.5%). Postoperative CT scans showed no evidence of intracerebral hemorrhage. An immediate postoperative improvement in motor symptoms was observed in all patients. Consequently, the LED was reduced by 25% (median; range: 0–88%) following surgery. Five of the thirteen patients (38.5%) presented with neuropsychiatric SE shortly (range: 0–9 days) after STN-DBS placement: hypomania, psychomotor slowing, VF decline, delirium, and disorientation were observed in two patients each, while impulsivity and impatience occurred in one patient each. Temporary pharmacological intervention was required in three patients (patient #3, #4, and #5) and one patient (#3) was additionally admitted to intensive care unit for one day due to delirium. SE persisted for over a year in patient #5 with gradually improvement, while SE resolved completely and rapidly in all other patients. For details see Table 1.

**Table 1:** Patient characteristics

group	patient	sex	age	symptom duration (years)	Hoehn & Yahr	interval baseline PET – follow-up PET (d)	interval surgery – follow-up PET (d)	baseline LED (mg/d)	LED reduction (%)	neuropsychiatric side effects
with SE	1	f	61	6	3	41	8	1111	25	hypomania
	2	m	61	nd	2	95	6	2100	88	psychomotor slowing, VF decline
	3	m	65	10	3	120	14	1925	0	delirium, VF decline, disorientation
	4	m	61	15	3	172	5	1148	78	psychomotor slowing
	5	m	59	7	3	141	16	1007	10	delirium, disorientation, hypomania, impatience, impulsivity
	median		61*	8.5	3	120	8	1536	25	
without SE	6	m	56	8	3	300	5	650	31	
	7	m	71	2	2	154	6	0	0	
	8	m	50	13	2	146	6	160	19	
	9	m	45	7	3	55	6	1053	76	
	10	f	58	7	3	159	7	525	0	
	11	f	44	2	2	792	6	1690	44	
	12	f	54	12	3	81	6	1775	62	
	13	m	60	9	2	8	7	450	0	
	median		55*	7.5	2.5	150	6	851	25	

Group comparison between patients with side effects (SE) and without SE using U-test statistics or χ^2^ test; * *p *< 0.05). f female, m male, LED Levodopa Equivalent Daily Dose, VF verbal fluency, nd no data

### PET imaging

**Fig. 1:**
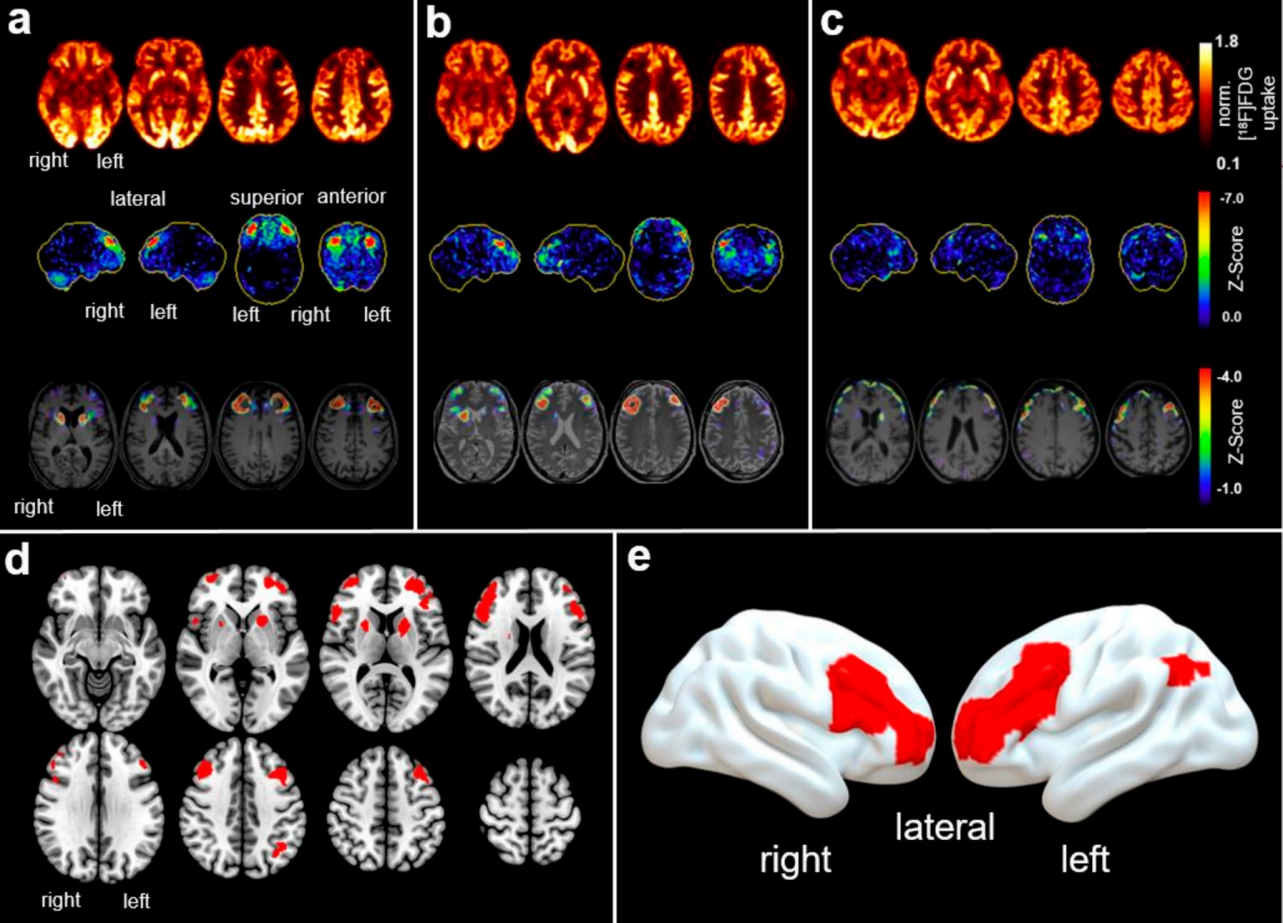
Glucose hypometabolism following electrode implantation. Panels **a-c** depict exemplary follow-up PET of patients with strongly (**a**), moderately (**b**) and mildly (**c**) expressed PET findings (upper row: transaxial slices of normalized [^18^F]FDG uptake; middle row: Neurostat/three-dimensional stereotactic surface projections [32] showing the *Z*-score based hypometabolism of the individual compared to a cohort of healthy controls; lower row: transaxial slices showing the *Z*-score based differences in regional normalized [^18^F]FDG uptake between baseline and follow-up PET overlayed to MRI). Panel **d** and **e** show results from voxel-wise group analysis with areas of reduced [^18^F]FDG uptake (red) of follow-up PET compared to baseline PET (*p* < 0.05 FDR corrected, *k*_*E*_ ≥ 100 voxels)

Follow-up PET revealed new bilateral frontal hypometabolism after DBS placement with positional reference to the electrodes in all patients (for exemplary findings see Fig. 1a-c). Voxel-wise analysis showed a significant decrease of regional glucose metabolism in the follow-up compared to baseline examinations (paired t-test, *p* < 0.001, *k*_*E*_ ≥ 100 voxels, all voxels surviving FDR correction at *p* < 0.05) (Fig. 1d-e). Large hypometabolic clusters were found in the frontal cortex of both hemispheres (left: *k*_E_ = 1718 voxels; right: *k*_E_ = 1550 voxels) and also in the right cerebellum (*k*_E_ = 1280 voxels). Other small clusters were localized in the left parietal cortex (*k*_E_ = 123 voxels) and in both striata (left: *k*_E_ = 374 voxels; right: *k*_E_ = 142 voxels).

**Fig. 2:**
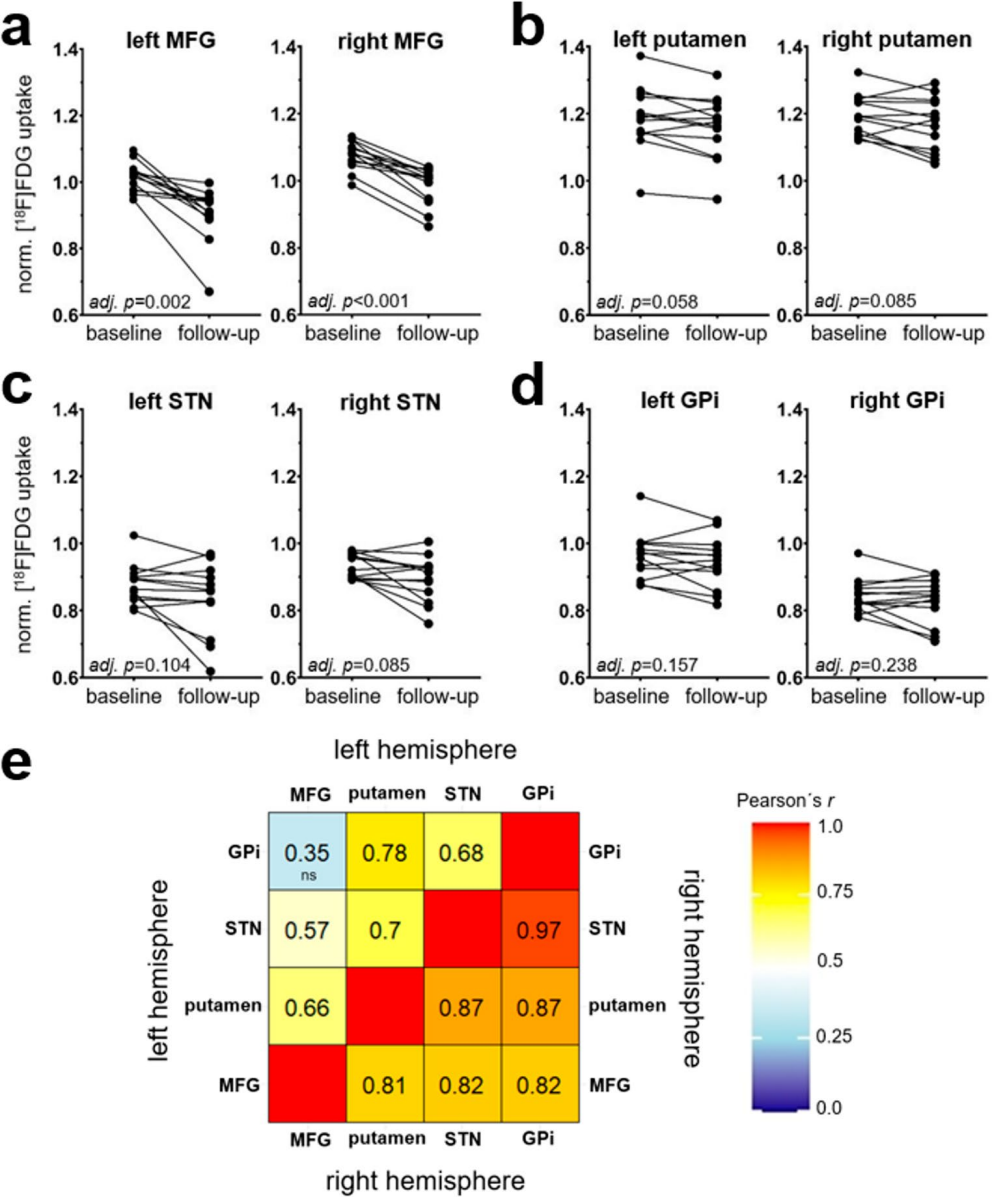
Paired dot plots and correlation matrix. Panels **a-d** shows the changes of normalized [^18^F]FDG uptake between baseline and follow-up PET in the (**a**) middle frontal gyrus (MFG), (**b**) putamen, (**c**) subthalamic nucleus (STN) and (**d**) globus pallidus internus (GPi). *p*-value adjusted (adj.* p*) with FDR-correction. Panel **e** shows the correlation of changes of normalized [^18^F]FDG uptake between baseline and follow-up PET in MFG, putamen, STN and GPi of the left hemisphere (upper left triangle) and right hemisphere (lower right triangle). Cells are shaded according to the strength of relationships based on Pearsons´s *r*. The correlations in the matrix were significant (*p* < 0.05 FDR-corrected), except for MFG ~ GPi in the left hemisphere, which was marked as not significant (ns)

VOI-based results corroborated the findings of the voxel-wise analysis. Normalized [^18^F]FDG uptake in the MFG of both hemispheres decreased highly significant from baseline to follow-up PET in all patients (left MFG: mean reduction 10.7 ± 8.1%, FDR adjusted *p* = 0.002, right MFG: mean reduction 8.4 ± 4.1%, FDR adjusted *p* < 0.001) (see also Fig. 2a; Table 2). The decrease of normalized [^18^F]FDG uptake in the putamen, STN and GPi was less pronounced (Fig. 2b-d) and did not reach statistical significance, though putamen and STN exhibited a trend-level effect (for details see Table 2). The percentage decrease of normalized [^18^F]FDG uptake exhibited a strong and significant correlation (FDR-corrected *p* < 0.05) among MFG, putamen, STN, and GPi in both hemispheres, and was more pronounced on the right. Only MFG and GPi in the left hemisphere did not correlate significantly (for details see Fig. 2e).

**Table 2:** Changes of normalized [^18^F]FDG uptake in predefined volumes-of-interest

VOI	baseline norm. [^18^F]FDG uptake (mean ± SD)	follow-up norm. [^18^F]FDG uptake (mean ± SD)	mean of difference (follow-up – baseline) [95% CI]	reduction (mean ± SD) (%)	adj.* p*-value	Cohens´s *d*
left MFG	1.02 ± 0.04	0.91 ± 0.08	−0.11 [−0.16 to −0.06]	10.7 ± 8.1%	0.002	1.36
right MFG	1.08 ± 0.04	0.99 ± 0.06	−0.09 [−0.12 to −0.06]	8.4 ± 4.1%	< 0.001	2.01
left putamen	1.19 ± 0.10	1.16 ± 0.09	−0.03 [−0.05 to −0.01]	2.2 ± 3.1%	0.058	0.73
right putamen	1.19 ± 0.06	1.17 ± 0.08	−0.03 [−0.05 to 0.00]	2.2 ± 3.8%	0.085	0.59
left STN	0.88 ± 0.06	0.83 ± 0.10	−0.04 [−0.09 to 0.01]	4.9 ± 9.2%	0.104	0.54
right STN	0.93 ± 0.04	0.90 ± 0.07	−0.04 [−0.07 to 0.00]	4.0 ± 6.4%	0.085	0.63
left GPi	0.96 ± 0.07	0.94 ± 0.08	−0.02 [−0.05 to 0.01]	2.0 ± 4.7%	0.157	0.44
right GPi	0.84 ± 0.05	0.83 ± 0.07	−0.02 [−0.04 to 0.01]	1.9 ± 5.6%	0.238	0.34

Results of paired t-tests comparing the differences of normalized [^18^F]FDG uptake in the middle frontal gyrus (MFG), putamen, subthalamic nucleus (STN) and globus pallidus internus (GPi) of both hemispheres between baseline and follow-up PET. *p*-value adjusted (adj.) with FDR-correction. VOI volumes-of-interest, norm. normalized, SD standard deviation, CI confidence interval

The mean extent of individual metabolic decrease (voxel-by-voxel subtraction; Z-score ≤ −2) were 2488 ± 1278 voxels (19.9 ± 10.2 ml) per hemisphere and 5137 ± 1092 voxels (41.1 ± 8.7 ml) in both hemispheres (for details see Supplementary Information 2). We detected a mean decrease of regional normalized [^18^F]FDG uptake along the electrode trajectory (magnitude of metabolic decrease) of −35.92 ± 18.04%, −29.32 ± 17.92% and − 23.40 ± 16.28%, in the small, medium and large VOI, respectively (for details see Supplementary Information 2). In individual patients, the decrease of regional normalized [^18^F]FDG uptake along the electrode trajectory in both hemispheres significantly declines with increasing VOI size (i.e., when also including grey matter located further away from the electrode) (one-way RM-ANOVA, left: F [2, 24] = 101.0, *p* < 0.001; right: F [2, 24] = 142.9, *p* < 0.001).

### Discriminating the onset of side effects

**Fig. 3:**
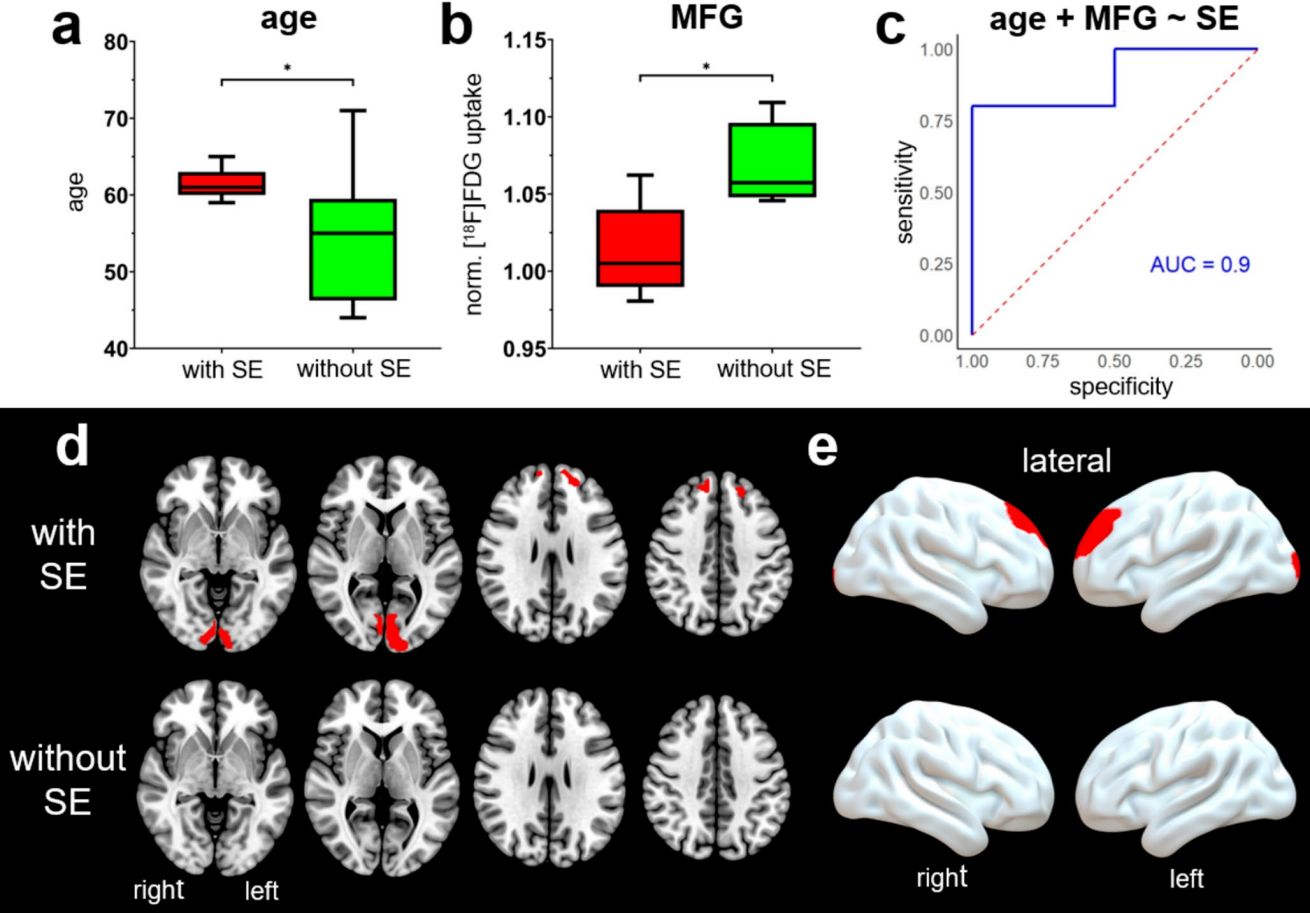
Association of side effects with age and baseline metabolism. Boxplots of (**a**) age and (**b**) normalized [^18^F]FDG uptake in the middle frontal gyrus (MFG) at baseline PET for groups with and without side effects (SE) (*p < 0.05; U-test). (**c**) Receiver operating characteristics curve for multivariate logistic regression model of patient´s age combined with metabolism in the MFG at baseline PET for the discrimination between patients with and without SE. (**d-e**) Voxel-wise group analysis shows areas of reduced [^18^F]FDG uptake (red) in the baseline PET of patients with (first row) and without SE (last row), compared to healthy controls (*p* < 0.001, *k*_*E*_ ≥ 100 voxels, peak voxels with *p* < 0.05 FDR corrected)

Patients with SE were older (median: 61 vs. 55 years; *p* = 0.043, *r*_rb_=0.70) and had lower normalized [^18^F]FDG uptake in the MFG at baseline PET compared to patients without SE (median: 1.01 vs. 1.06; *p* = 0.030, *r*_rb_=0.75) (Table 1; Fig. 3a-b). All other demographic and clinical characteristics (sex, symptom duration, Hoehn & Yahr stage, LED reduction) and image-based variables (extent and magnitude of metabolic decrease) did not show significant differences between the groups (Table 1 and Supplementary Information 2).

The discriminatory power of the significant parameters calculated by the ROC AUC yielded 0.88 for MFG metabolism and 0.85 for age. When combining both independently significant parameters in a multivariate logistic regression model, only MFG metabolism showed a trend toward significance (*p* = 0.091), while age was not significant (*p* = 0.812). The model yielded an ROC AUC of 0.90 (Fig. 3c). Voxel-wise group comparison of baseline PET with healthy controls revealed no significant differences in regional cerebral metabolism in patients without SE. In contrast, patients with SE showed significantly reduced metabolism in bilateral frontal (left: *k*_E_ = 241 voxels; right: *k*_E_ = 152 voxels) and occipital (left: *k*_E_ = 563 voxels; right: *k*_E_ = 239 voxels) regions compared to healthy controls (two sample t-test, *p* < 0.001, *k*_*E*_ ≥ 100 voxels, peak voxels surviving FDR correction at *p* < 0.05) (Fig. 3d-e).

## Discussion

The present study investigates cerebral metabolic changes in advanced PD patients in the early stage after STN-DBS surgery (up to 16 days, median 6 days) by systematically evaluating early follow-up [^18^F]FDG-PET scans without active stimulation in comparison to baseline scans before surgery. The results demonstrate a significant metabolic decrease in frontal cortical and subcortical areas along the electrode trajectories after surgery. We found no association of regional metabolic changes with neuropsychiatric (including cognitive) SE. The latter instead, were associated with age and metabolism in the MFG at baseline prior surgery. This suggests that pre-existing cortical degeneration in PD predisposes patients to neuropsychiatric SE following STN-DBS surgery.

Voxel-wise comparison of baseline and follow-up PET revealed bilateral metabolic decrease in the frontal cortex and the striatum (Fig. 1d-e), which was confirmed by the VOI analysis (i.e., significant hypometabolism in MFG, putaminal hypometabolism at trend level (Fig. 2a-b)). The mean metabolic decrease in both hemispheres was 41.1 ± 8.7 ml. This represents an involvement of at least 10% of the total frontal lobe volume, which corresponds to about 387.2 ml in healthy controls [33]. Furthermore, extent and magnitude of metabolic decrease did not show any association with findings of the contralateral side (data not shown), suggesting a direct effect from the respective insertion of the electrode. Other clusters of hypometabolism at follow-up may be attributed primarily to diaschisis (right cerebellum) or, given the small size, possibly also non-specific effects/noise (left parietal cortex). With increasing distance from the electrodes, which was mimicked by the different size of the VOI defined around the electrode, the magnitude of metabolic decrease significantly declined. Together with the strong correlation of metabolic decrease between MFG, putamen and STN within a hemisphere (Fig. 2e), this indicates that the electrode insertion was the epicenter and likely cause of neuronal dysfunction. Alternative explanations like network-mediated effects projecting onto cortical regions elicited by MLE only within the STN are less plausible, which is in line with studies suggesting that additional lesions in (sub)cortical structures along the electrode trajectory occur and contribute to SE [11, 17]. Nevertheless, network-mediated effects originating at the subcortical level cannot be entirely excluded. Given that the GPi is not expected to be directly affected by the electrode trajectory, the correlated metabolic decrease in the STN and GPi may reflect MLE-inhibited projections from the STN to the GPi, potentially explaining a part of the motor improvement. It would be intriguing to compare these findings with the emerging technique of magnetic resonance-guided focused ultrasound subthalamotomy [34], which provides a less invasive approach and may reveal solely network-mediated effects on the GPi.

To our knowledge, surgery-related frontal hypometabolism in the early phase after STN-DBS has not been assessed before, as follow-up [^18^F]FDG-PET of previous studies has been performed at three months or later after surgery [35, 36]. Partly because the studies mainly focused on the effect of active stimulation, the available data is inconclusive. Whereas, without active stimulation, no metabolic differences compared to baseline were observed four months after surgery [35], a decrease of metabolism in the STN was observed six months after STN-DBS placement and considered to originate from remaining small brain lesions due to the electrode [36]. Furthermore, [^18^F]FDG-PET studies with patients who received DBS due to other diseases did not report hypometabolism alongside the electrodes at medium- or long-term follow-up [37, 38], which is in line with our clinical experience of PD and other diseases treated with DBS. Thus, since the hypometabolism in the frontal cortex and subcortical structures does not persist, a temporary neuronal dysfunction instead of cortical damage can be assumed. Only few functional MRI studies examined early postoperative changes without stimulation compared to the preoperative condition, showing altered connectivity in motor and brainstem networks [39, 40]. However, complementary data regarding neuropsychiatric outcomes are lacking but would be valuable to better understand the underlying mechanisms of transient postoperative dysfunction.

The occurrence of neuropsychiatric (including cognitive) SE after DBS has been reported in several studies and meta-analyses. Due to varying study designs (i.e. differences in SE reporting or follow-up intervals) the reported incidence of SE differs. In our cohort, two patients developed one and two SE respectively, while one patient showed five SE (Table 1). However, the combined 38.5% incidence of neuropsychiatric SE observed in our cohort is consistent with the reported incidence of individual neuropsychiatric symptoms, including 4–15% for (hypo)mania, 15.4% for speech disorders, 11.9% for delirium, 22.7% for obsessive-compulsive disorders, and 12.8% for severe psychiatric SE, whereby longer intervals after surgery and active stimulation were also included [12, 13, 41].

Contrary to our hypothesis, we observed no association between the magnitude and extent of hypometabolism and the occurrence of neuropsychiatric SE. Similarly, there were no differences in sex, symptom duration and Hoehn & Yahr stage between patients with and without SE. However, patients with SE had a lower baseline metabolism in the MFG and were older (Fig. 3a-b). When combining these two parameters in a multivariate logistic regression model, the derived ROC AUC showed a good separation between the groups (Fig. 3c). Although at a statistical trend level only, this finding suggests that possible, pre-existing degeneration of the frontal lobe (as reflected by PET) could be a risk factor for neuropsychiatric SE after DBS surgery. Along with disease progression, PD-related pathology spreads to higher and first order association areas of the neocortex and premotor areas [42]. This cortical involvement in advanced PD is reflected by glucose hypometabolism, which can be detected predominantly in the posterior temporoparietal and occipital regions but also in the frontal lobe [24]. In our cohort cortical involvement prior to DBS surgery could be detected in patients with SE, but not in patients without SE, when compared at group level to healthy controls (Fig. 3d-e). Since such hypometabolism may precede cognitive decline [43], it is conceivable that patients with an incipient frontal degeneration (not yet paralleled by clinical symptoms) are already sensitive to the additive temporary implantation alterations observed in the current study. Although such a frontal degeneration is more likely associated with PD–related pathology there exist other possibly contributing factors, like Alzheimer’s disease-related copathology, mild vasculopathy or age since older age is intrinsically associated with a certain degree of frontal degeneration [44].

The analysis of cerebral [^18^F]FDG-PET for such a risk stratification on the short-term outcome expands its current diagnostic and prognostic utility prior the use of DBS. PET is powerful tool for differential diagnosis of parkinsonism, e.g. to rule out atypical parkinsonian syndromes before DBS [24]. Moreover, a recent study showed the prognostic value of cerebral cortical glucose metabolism for overall survival in patients with Lewy Body Diseases [45]. Based on the present study, available PET scans could also be evaluated regarding frontal hypometabolism and taken into account for the treatment decision and, if necessary, postoperative therapy and care concepts.

Both the retrospective study design and the small number of included patients are limitations of the present study. There is some variability in the time between baseline, surgery and follow-up, which could have affected the results and group comparisons when considering that postoperative hypometabolism and MLE are likely transient. Moreover, the retrospective nature leads also to an insufficiently systematic collection of clinical variables, in particular neuropsychological parameters and motor response. Given that [^18^F]FDG-PET was performed for clinical reasons and patients were not consecutively enrolled, the requirement of an early follow-up PET may have introduced a selection tendency toward patients who developed neuropsychiatric SE after DBS surgery. Although previous work with small brain nuclei showed reliable results [26, 27], it should be noted that partial volume error may have affected the quantification particularly of the STN and thus the analysis with predefined VOI. Moreover, the two parameters (baseline MFG metabolism and age) did not reach the significance level in the multivariate logistic regression model, which we attribute to the small sample size. Nonetheless, the model demonstrated good discriminative ability (AUC = 0.90). Therefore, and for the definition of cut-off values, it is necessary to prospectively validate our findings within a larger cohort. Furthermore, the grouping of several neurological, psychiatric and cognitive symptoms under the term neuropsychiatric SE is certainly simplistic and limiting, as these symptoms likely reflect different pathophysiological mechanisms. A separation into individual symptoms or symptom complexes in further studies may provide additional insights. Future studies should also consider the dynamics of neuropsychiatric SE over time, as they may regress, whereas predictors of persistence are of particular clinical relevance.

## Conclusion

Cortical and subcortical neuronal dysfunction along the electrode trajectory early after STN-DBS can be detected by [^18^F]FDG-PET and suggests transient surgery-induced effects. Baseline metabolism in the MFG and age were associated with neuropsychiatric side effects after surgery supporting the preoperative use of PET to enhance DBS safety by improving patient selection and postoperative care concepts.

## Supplementary Information

Below is the link to the electronic supplementary material.

Supplementary Material 1 (DOCX. 1.00 MB)

## Data Availability

The data from this study are available from the corresponding author on reasonable request.
