# Supplementary material for: [18F]FDG-PET reveals early postoperative cortical dysfunction after subthalamic nucleus deep brain stimulation in Parkinson’s disease
Source: Eur J Nucl Med Mol Imaging. 2025 Oct 2;53(3):1980–90. doi: 10.1007/s00259-025-07552-0 (PMC12860824; doi:10.1007/s00259-025-07552-0)
Supplement: Supplementary file 1 — Supplementary Material 1 (DOCX. 1.00 MB) [file 259_2025_7552_MOESM1_ESM.docx]

**Supplementary Information**

**Article title:**

[^18^F]FDG-PET reveals early postoperative cortical dysfunction after subthalamic nucleus deep brain stimulation in Parkinson's disease

**Authors:**

Christian Volz^1^, Volker A. Coenen^2^, Lars Frings^1^, Michel Rijntjes^3^, Horst Urbach^4^, Philipp T. Meyer^1^, Bastian E. A. Sajonz^2#^, Joachim Brumberg^1#^

^#^These authors contributed equally

^1^Department of Nuclear Medicine, Medical Center – University of Freiburg, Faculty of Medicine, University of Freiburg, Freiburg, Germany

^2^Department of Stereotactic and Functional Neurosurgery, Medical Center – University of Freiburg, Faculty of Medicine, University of Freiburg, Freiburg, Germany

^3^Department of Neurology and Clinical Neuroscience, Medical Center – University of Freiburg, Faculty of Medicine, University of Freiburg, Freiburg, Germany

^4^Department of Neuroradiology, Medical Center – University of Freiburg, Faculty of Medicine, University of Freiburg, Freiburg, Germany

**Journal name:**

European Journal of Nuclear Medicine and Molecular Imaging

**Corresponding Author:**

Dr. Christian Volz

Department of Nuclear Medicine, Medical Center – University of Freiburg

Faculty of Medicine, University of Freiburg,

Hugstetter Straße 55, 79106 Freiburg, Germany

ORCID: 0009-0006-5464-5607

Mail: christian.volz@uniklinik-freiburg.de

**
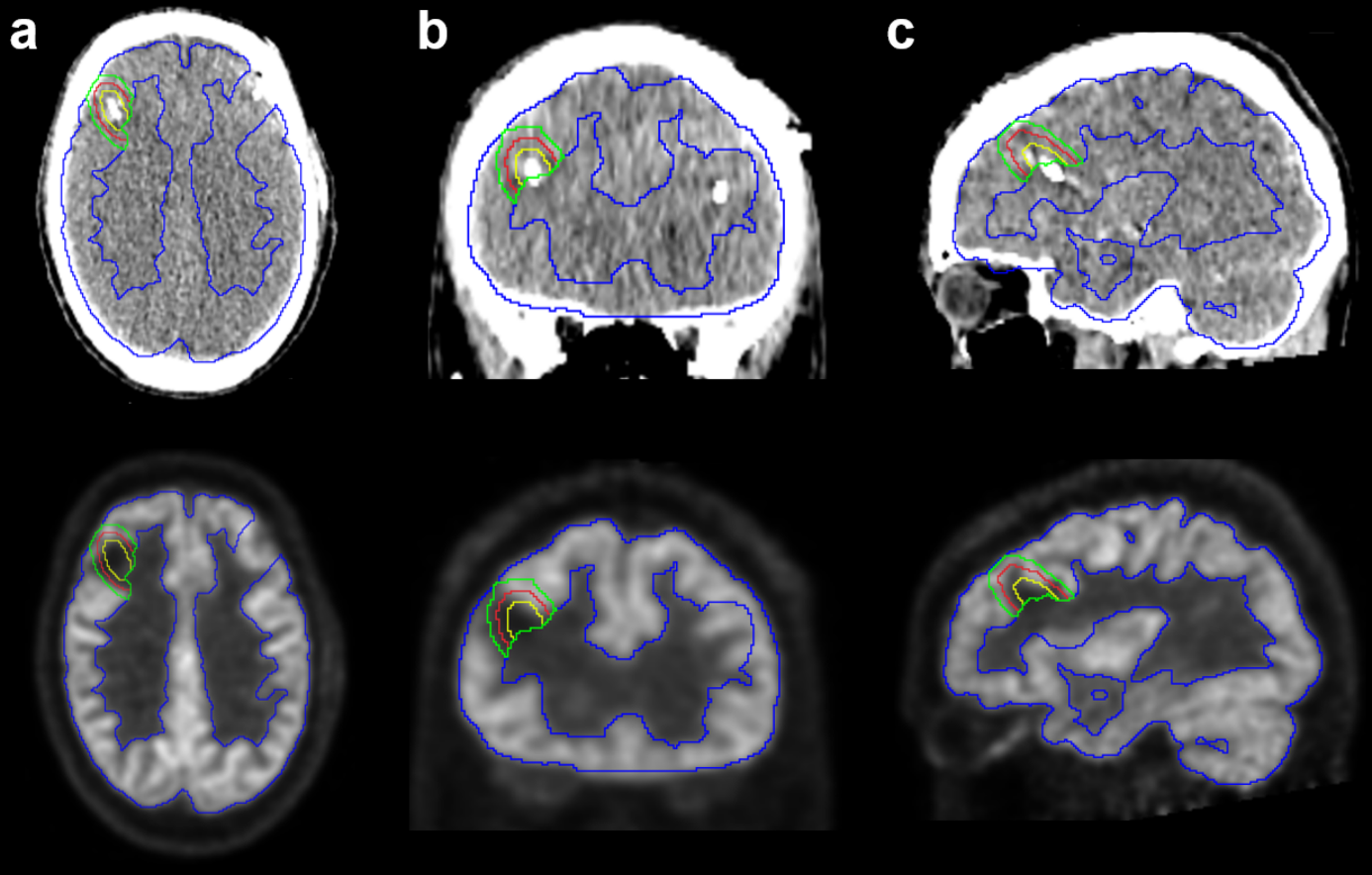
Supplementary Information 1 Example of trajectory** **and** **adjusted grey matter VOI** Trajectory VOI of the right hemisphere and adjusted grey matter VOI overlayed on low-dose CT (top row) and follow-up [^18^F]FDG-PET (bottom row). VOI were manually generated as previously described. Images are displayed in the individual´s space in (a) transaxial, (b) coronal and (c) sagittal view. Blue contour, adjusted grey matter VOI; green contour, large trajectory VOI; red contour, medium trajectory VOI; yellow contour, small trajectory VOI.

**Supplementary Information 2** **Magnitude and extent of metabolic decrease**

| group |  | right hemisphere metabolic decrease | | | | left hemisphere metabolic decrease | | | |
| --- | --- | --- | --- | --- | --- | --- | --- | --- | --- |
|  | patient | extent (ml) | magnitude (change of norm. [^18^F]FDG uptake) | | | extent (ml) | magnitude (change of norm. [^18^F]FDG uptake) | | |
|  |  |  | small trajectory VOI | medium trajectory VOI | large trajectory VOI |  | small trajectory VOI | medium trajectory VOI | large trajectory VOI |
| with SE | 1 | 23.7 | -12.32 % | -6.96 % | -4.11 % | 22.5 | -24.67 % | -15.69 % | -9.51 % |
|  | 2 | 18.6 | -56.26 % | -53.05 % | -45.88 % | 19.3 | -72.92 % | -64.25 % | -52.96 % |
|  | 3 | 33.4 | -42.52 % | -37.05 % | -28.78 % | 3.9 | -10.33 % | -4.50 % | -1.88 % |
|  | 4 | 10.1 | -16.94 % | -8.45 % | -6.39 % | 42.9 | -70.16 % | -65.77 % | -59.40 % |
|  | 5 | 16.1 | -18.92 % | -15.86 % | -11.70 % | 19.3 | -31.45 % | -27.22 % | -23.07 % |
|  | median | 18.6 | -18.92 % | -15.86 % | -11.70 % | 19.3 | -31.45 % | -27.22 % | -23.07 % |
| without SE | 6 | 31.0 | -29.33 % | -20.06 % | -14.30 % | 15.6 | -24.10 % | -16.66 % | -11.02 % |
|  | 7 | 11.3 | -20.58 % | -15.69 % | -12.12 % | 38.3 | -43.17 % | -34.42 % | -26.31 % |
|  | 8 | 9.6 | -36.84 % | -30.13 % | -22.62 % | 32.9 | -54.79 % | -51.69 % | -46.51 % |
|  | 9 | 29.4 | -53.77 % | -44.66 % | -36.59 % | 9.8 | -25.61 % | -17.70 % | -13.27 % |
|  | 10 | 16.8 | -36.83 % | -30.54 % | -22.29 % | 16.0 | -32.85 % | -22.62 % | -16.26 % |
|  | 11 | 23.2 | -63.64 % | -56.53 % | -47.93 % | 20.9 | -55.38 % | -45.27 % | -37.43 % |
|  | 12 | 10.1 | -26.50 % | -17.62 % | -12.48 % | 18.1 | -32.70 % | -26.13 % | -19.25 % |
|  | 13 | 23.5 | -29.40 % | -24.51 % | -19.84 % | 1.4 | -11.84 % | -9.28 % | -6.52 % |
|  | median | 20.0 | -33.12 % | -27.32 % | -21.07 % | 17.1 | -32.78 % | -24.38 % | -17.76 % |

Group comparison between patients with side effects (SE) and without SE using U-test statistics (*p*<0.05); no significant differences. VOI, volume of interest.
